# Palbociclib as an Antitumor Drug: A License to Kill

**DOI:** 10.3390/molecules29225334

**Published:** 2024-11-13

**Authors:** Agnieszka Łupicka-Słowik, Federica Cossu, Marcin Sieńczyk

**Affiliations:** 1 Division of Organic and Medicinal Chemistry, Faculty of Chemistry, Wroclaw University of Science and Technology, Wybrzeze Wyspianskiego 27, 50-370 Wroclaw, Poland; marcin.sienczyk@pwr.edu.pl; 2 National Research Council, Institute of Biophysics (IBF-CNR), Milan Unit, Via Corti, 12, 20133 Milan, Italy; federica.cossu@cnr.it

**Keywords:** palbociclib, antitumor, TNBC, targeted therapy, PROTAC, SNIPERS, cyclin-dependent kinases

## Abstract

Neoplastic cells are characterized by uncontrolled cell divisions caused by cell cycle dysregulation. Key regulatory proteins governing the transition from the G1 to the S phase are the CDK4 and CDK6 kinases, which are controlled by D-type cyclins. The CDK4/6 kinases enable the use of these proteins as targets for anticancer therapy because they prevent the growth and the development of malignant cells by inhibiting their activity. This paper surveys the clinical trial results concerning palbociclib, the first in-class FDA-approved anticancer drug for hormone-dependent breast cancer. It discusses the therapeutic applications in breast cancer as well as in solid tumors and hematopoietic malignancies. Additionally, the paper presents an analysis of palbociclib resistance acquired during therapy and explores new approaches, such as modifications to palbociclib that enhance its desired activity or open up new therapeutic possibilities (PROTACs).

## 1. Introduction

**Figure 1:**
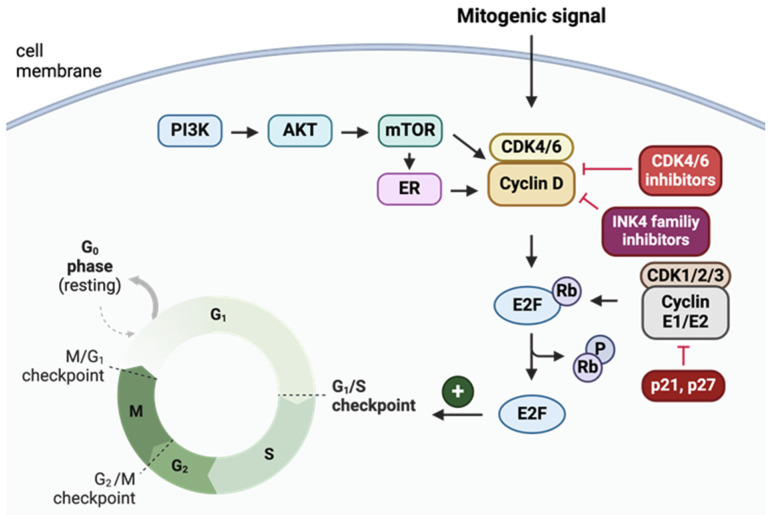
General mechanism of action of CDK4/6 in cell cycle control. AKT—protein kinase B, CDK4/6—cyclin dependent kinase 4 and 6, E2F—transcription factor family, ER—estrogen receptor, mTOR—mammalian target of rapamycin, PI3K—phosphoinositide 3-kinase, and Rb—retinoblastoma [2]. Created in BioRender. Sienczyk, M. (2024); https://BioRender.com/z65l923 (accessed on 6 November 2024).

The cell cycle is regulated mainly by complexes of cyclin D–CDK (cyclin-dependent kinases) that act as checkpoints in the transition from G1 phase (cell growth) to S (DNA synthesis) phase (DNA damage checkpoint). It is a disturbed cell cycle that causes abnormal and uncontrolled cell divisions in neoplastic cells. CDK4 and CDK6, governed by D-type cyclins, are the key mediators responsible for the cellular progression from G1 to S phase. They are highly homologous proline-binding serine/threonine protein kinases (70% of amino acid identity, 80% positives according to the BLAST alignment [1] of CDK4 UniProt ID: P11802 and CDK6 UniProt ID: Q00534). Activated holoenzyme cyclin D–CDK4/6 phosphorylates a wide range of substrates, including the retinoblastoma suppressor protein (Rb) that facilitates the release of transcription factors followed by further transcription of the S phase genes (Figure 1) [2].

The second canonical mechanism behind G1 to S phase progression is based on the sequestration of CDK inhibitors p21 and p27 that are responsible for binding and preventing cyclin E–CDK2 activity (which also phosphorylates Rb, thus facilitating tumorigenesis) [3].

Cyclin D–CDK4/6 activation plays a role in initiating the growth and survival of various cancer cells [4,5]. In many tumors, the expression levels of CDK4/6 are significantly elevated (hematopoietic malignancies, breast cancer, and melanoma), which makes the CDK4/6 inhibitors attractive as therapeutic targets [6]. The activity of CDK4/6 is endogenously inhibited by binding the INK4 family inhibitors (p16INK4A, p15INKB, p18INK4C, and p19INK4D; Figure 1), preventing the phosphorylation of Rb [7]. Inhibition can also be intentionally established by low molecular weight inhibitors, such as palbociclib, ribociclib, or abemaciclib [8].

## 2. Palbociclib as an Antitumor Drug

### 2.1. CDK4/6 as Targets for Anticancer Therapy

During the cell cycle, cyclins activate CDKs in order to progress to the next stage [9]. While the CDKs’ levels remain stable throughout the cycle, their subcellular concentrations change considerably. Such variations selectively activate particular CDKs under the control of cyclins [10]. It is the catalytic activity of CDKs that is more important for cell cycle progression than their specificity and cellular localization [11,12]. Zhang et al. [11] performed a detailed in silico analysis that compares the catalytic activity potency of the active cyclin D–CDK4 and active cyclin E–CDK2 complexes. The active complex of cyclin D–CDK4 was found to have a more flexible ATP binding site, displayed weaker hydrogen bonds between ATP and CDK4, and showed an allosteric regulation of the cyclin D N-terminus, which resulted in lower catalytic competence [11]. The activity of CDK4/6 kinases is regulated at the transcriptional level by their intracellular localization and by their association with cyclin D. The association of CDK4/6 with cyclins is regulated by the INK4 proteins (including p15INK4a, p16INK4a, p18INK4a, and p19INK4a) that diminish their interaction with cyclin D and interact with the catalytic domains of CDK4/6.

CDK4 and 6 enable the E2F transcription factor, which is crucial for the G1/S transition (Figure 1). Their inhibition could arrest the cell cycle in the G1 phase [9]. In neoplastic cells, mutations of these crucial proteins are common. Thus, for example, the overexpression of CDK4/6 that enhances the G1/S transition directly and indirectly (through stimulation of CDK2 that initiates the positive feedback loop) leads to the phosphorylation of Rb. Inhibition of the CDK4/6 activity is also beneficial for tumor cells that lack the Rb protein, leading to apoptosis or a delay of the G1/S transition [13].

**Figure 2:**
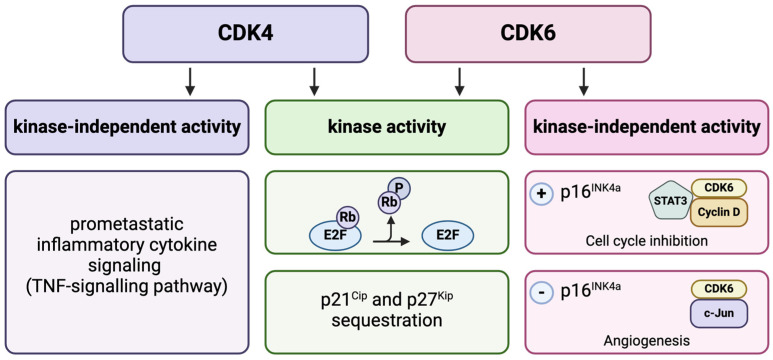
The significance of kinase-independent activities of CDK4/6 kinases. Based on [14,15]. Created in BioRender. Sienczyk, M. (2024); https://BioRender.com/j69m843 (accessed on 6 November 2024).

There are also distinct cell cycle-independent functions of CDK4 and 6 that promote the development and progression of cancer (Figure 2). Dai et al. [14] revealed that CDK4 can regulate tumor growth by affecting the inflammatory cytokine signaling, whereas CDK6 controls the DNA replication and repair processes. The kinase-independent function of CDK6 is the upregulation of p16INK4a, which, in the case of the overexpression of CDK6, suppresses the proliferation of the cell (internal safeguard). Importantly, in the absence of p16INK4a, CDK6 accelerates and promotes angiogenesis and enhances proliferation through the transcription of growth factors, such as Vegf-A [15].

The presence of CDK4/6 kinases in cell management can be utilized in antitumor therapy. The cyclin D–CDK4/6 complexes are involved in tumor formation and metastasis in various solid tumors, including those of the breast [16,17], pancreas [18], and prostate [19]. A significant role of CDK4/6 kinases was reported in hematopoietic cancers, such as acute lymphoblastic leukemia [20] or mantle cell lymphoma [21].

**Figure 3:**
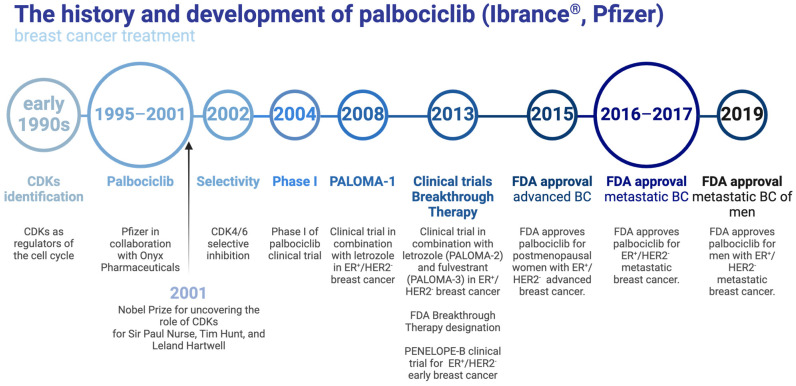
The history and development of palbociclib based on Pfizer datasheets. Created in BioRender. Sienczyk, M. (2024); https://BioRender.com/o20f315 (accessed on 6 November 2024).

Anticancer therapy targeting the CDK4/6 kinases can be directed toward inhibition of their enzymatic activity, thus preventing the growth and the development of malignant cells. The first-generation CDK inhibitors with limited clinical application showed low specificity (pan-CDK) and therefore led to numerous adverse side effects. The most extensively investigated compound (more than 60 clinical trials) is alvocidib (flavopiridol, developed by Sanofi-Aventis, Bridgewater, USA) that blocks CDK1/2/4/6/7/9 [22,23]. Another ATP-competitive inhibitor of cyclin-dependent kinases, abemaciclib (Verzenios^®^, LY2835219; Eli Lilly, Indianapolis, IN, USA), is capable of inhibiting CDK4/6 (with greater selectivity for CDK4 over 6) and CDK9. Abemaciclib received an FDA Breakthrough Therapy designation in 2015. In phase I first-in-human clinical trial, abemaciclib activity was initially investigated in patients with five advanced tumor types: NSCLC, glioblastoma, breast cancer, and colorectal cancer (ClinicalTrials.gov ID: NCT01394016). Another study (MONARCH 2 and 3, ClinicalTrials.gov ID: NCT02107703 and NCT02246621, respectively) dealt with a combination of abemaciclib with other active compounds (fulvestrant and non-steroidal aromatase inhibitors) in breast cancer. Ribociclib (Kisqali^®^, LEE011; Novartis, Basel, Switzerland) and palbociclib (Ibrance^®^, PD-0332991; Pfizer, New York, NY, USA) are characterized by higher selectivity towards CDK4/6 compared to abemaciclib. In 2016, as a result of the Phase III Monaleesa-2 trial (ClinicalTrials.gov ID: NCT01958021), the FDA granted ribociclib a breakthrough therapy status in combination with letrozole for hormone receptor positive (HR+) and human epidermal growth factor 2 negative (HER2-) advanced or metastatic breast cancer. Palbociclib, also considered a breakthrough therapy, was the first CDK4/6 inhibitor approved by the FDA (Figure 3, 2015). Its effectiveness was examined, among others, in randomized PALOMA clinical trials in combination with letrozole (phase II PALOMA-1, ClinicalTrials.gov ID: NCT00721409, phase III PALOMA-2, ClinicalTrials.gov ID: NCT01740427) and fulvestrant (phase III PALOMA-3, ClinicalTrials.gov ID: NCT01942135), which is especially focused on breast cancer patients. Currently, a number of clinical trials investigate various settings of these CDK4/6 selective inhibitors in combination with other targeted antitumor agents.

Asghar et al. [23] reported that these three specific inhibitors bind to the CDK4/6 ATP-binding pocket. Currently, there are structural data of cocrystal structures as determined by Chen et al. [24], indicating the binding modes for abemaciclib (PDB ID: 5L2S), ribociclib (PDB ID: 5L2T), and palbociclib (PDB ID: 5L2I). The crucial structural element determining the selectivity of CDK inhibitors is the interaction of particular inhibitors with the non-conserved regions of the ATP-binding pocket [24].

CDK inhibitors are not only useful for anticancer therapy but also protect normal cells from cancer therapy-induced toxicity. Myelopreservation and resistance to chemotherapy-induced damage were observed during parallel administration of trilaciclib (COSELA^®^; G1 Therapeutics, New York, NY, USA) in small-cell lung cancer patients [25,26].

A novel concept of designing drugs targeting CDK4 proposed by Zhang et al. [11] relies on the fact that the CDK4 ATP binding site is regulated by the N-terminus of cyclin D. In this strategy, an allosteric inhibition stabilizes the CDK4–cyclin D complex in its inactive form [11].

CDK4/6 inhibitors are most potent when a specific aberration of cell cycle occurs in particular neoplastic cells. For this reason, details about the nature of neoplastic changes are needed, especially in the early stages of the disease. Although some tumors are initially susceptible to CDK4/6 inhibitors, they may become insensitive to these therapies over time. This insensitivity could be triggered by, e.g., upregulating CDK6, suggesting the necessity of developing stronger or more specific inhibitors and alternative strategies. Another important feature of CDK6 is its kinase-independent activity, which is why silencing the protein is more valuable than inhibition of its activity [14,15,27,28]. Thus, the development of a novel strategy against CDK4/6-centered malignancies is of great interest and importance. One of these strategies may be the application of proteolysis targeting chimeras (PROTACs) [13]. New opportunities given by PROTACs enable E3 ligase to ubiquitinate the protein of interest (POI) targeted by a specific binder followed by proteasomal degradation, overcoming the drug resistance through complete elimination of the POI.

### 2.2. Palbociclib in Clinical Trials

More than 300 clinical trials for more than 50 tumor types investigated the antitumor activity of palbociclib, ribociclib, or abemaciclib—three small-molecule CDK4/6 inhibitors. The clinical trial database (Clinicaltrials.gov, National Library of Medicine, National Center for Biotechnology Information) shows 378 clinical trials featuring the keyword palbociclib. These compounds demonstrated benefits (as monotherapy or in combination) in clinical studies, particularly in breast cancer (most beneficial estrogen-receptor positive; ER^+^), but also in melanoma, mantle cell lymphoma, non-small-cell lung cancer, and head and neck squamous cell carcinoma [29].

#### 2.2.1. Breast Cancer

Breast cancer (BC) is classified into four categories based on the expression of hormone receptors (HRs): estrogen receptor positive (ER^+^), progesterone receptor positive (PR^+^), and human epidermal growth factor receptor positive (HER2^+^). The fourth category refers to triple-negative breast cancer (TNBC, basal-like) that does not express any of the indicated receptors. Among these four types, TNBC is a highly metastatic and the most aggressive BC subtype with a poor prognosis [30]. BC can also be classified as luminal A (HR^+^/HER2^−^) and B (HR^+^/HER2^+^) depending on receptor expression, which constitutes 70% of the cases. Treatment of BC positive for hormone receptors takes advantage of ER antagonists and aromatase inhibitors. It is also possible to utilize immunotherapy directed to the HER2 receptors in the HER2^+^ subtype. TNBC, due to its immunochemical profile, requires a different therapeutic approach [31,32].

Preclinical data show palbociclib’s ability to inhibit the growth of particular subgroups of breast cancer cells synergistically with ER antagonists. It was possible to identify the criteria for patient selection in clinical studies with palbociclib [16]. This research was the starting point for the clinical trial PALOMA-1 (open-label, randomized, proof-of-concept study) that showed significantly longer progression-free survival for letrozole (an aromatase inhibitor, standard treatment for postmenopausal women with HR^+^/HER2^-^ breast cancer) in combination with palbociclib than letrozole alone [33]. As a continuation/confirmation of PALOMA-1, the PALOMA-2 clinical trial (Table 1) was designed. The PALOMA-2 clinical trial was designed for postmenopausal women with advanced HR^+^/HER2^-^ breast cancer. This study showed that palbociclib as an additive to standard endocrine therapy could significantly improve the treatment outcomes. The median progression-free survival (PFS) in PALOMA-2 compared to other phase III studies was longer: it was 24.8 months for the palbociclib–letrozole group and 14.5 months for the placebo–letrozole group. The most common adverse events were neutropenia (grade 3 or higher, occurring in 66.4% and 1.4% of the palbociclib–letrozole and placebo–letrozole groups, respectively), leukopenia (24.8% vs. 0%), and anemia (5.4% vs. 1.8%) [34]. The PALOMA-2 study was complemented by the PALOMA-4 clinical trial that included an Asian women group (Table 1). The median PFS obtained in this clinical trial was 21.5 and 13.9 months for the palbociclib–letrozole and placebo–letrozole groups, respectively. In PALOMA-4, similar adverse effects were recorded: neutropenia (84.5% vs. 1.2%), leukopenia (36.3% vs. 0.6%), thrombocytopenia (6.5% vs. 0.6%), and anemia (4.8% vs. 1.8%) [35].

Because PALOMA-1/2 studies did not include Japanese women, a phase I/II study of the efficacy of palbociclib in this population was conducted (NCT 01684215, Table 1). The median PFS in this trial was 35.7 months, and the objective response rate was 47.6%. Grade 3/4 neutropenia, leukopenia, and stomatitis were reported as common adverse events related to the treatment [37]. Also, a population of postmenopausal women from Mexico and selected Latin American countries with HR^+^/HER2^−^ breast cancer for whom letrozole was an applicable therapy participated in phase III clinical trial (NCT02600923, Table 1) to evaluate the efficacy of palbociclib. The objective response rate was 24.8%, and the palbociclib–letrozole combination was generally well tolerated with adverse effects similar to those reported previously [39].

After the registration of palbociclib therapy for ER^+^/HER2^−^ ABC in the USA (2015), a phase IV interventional study was performed for postmenopausal women with HR^+^/HER2^−^ eligible for letrozole treatment in Australia and India (NCT02679755, Table 1). The treatment was well tolerated and manageable, despite the safety profile consistent with previous reports. The objective response rates based on the evaluation of the investigator were 19.4% in the overall population, which is a lower value than in the first-line PALOMA studies (43.0% PALOMA-1 and 42.1% PALOMA-2). This difference may be due to different previous treatment of the patients (PALOMA 1/2 participants did not receive previous systemic treatment for ABC) or differences in the population of the patients, such as the median age [40].

PALLET (Table 1) is an example of a clinical trial carried out to evaluate the combination palbociclib–letrozole as a neoadjuvant therapy in patients with early ER^+^ breast cancer. The therapy of letrozole in combination with palbociclib enhanced the suppression of the proliferation of malignant cells when compared to letrozole alone; however, this did not increase the expected shrinkage of the tumor in ultrasound imaging. Similar to the previously described trials, neutropenia turned out to be an important issue related to therapy toxicity [38].

A phase II PALTAN study (Table 1) involved patients with ER^+^/HER2^+^ breast cancer for whom immunotherapy-induced endocrine therapy is the treatment of choice. This trial evaluated neoadjuvant combination therapy with palbociclib–letrozole–trastuzumab (PLT). Despite the fact that the therapy was feasible and well tolerated, the trial was terminated due to a low complete pathological response rate (7.7%) [41].

HR^+^ breast cancer patients with histologically confirmed invasive lobular carcinoma or invasive ductal carcinoma were enrolled in the PELOPS clinical trial (Table 1). The study had two objectives: to determine the difference in the antiproliferative activity in the letrozole–tamoxifen vs. tamoxifen therapy and to determine the complete pathological response rate of the endocrine therapy vs. palbociclib–endocrine therapy. The percentage of participants with a clinical response rate according to the results presented in the outcome measures were 57.8% and 43.3% for palbociclib–letrozole and letrozole, respectively.

Palbociclib–letrozole or palbociclib–fulvestrant as a therapy for patients over 70 years of age with metastatic HR^+^/HER2^−^ breast cancer was evaluated in a clinical trial (NCT03633331, Table 1) evaluating the tolerability of these combinations.

As part of the clinical trials conducted under the PALOMA acronym, PALOMA-3 was intended for evaluation therapy for HR^+^/HER2^-^ advanced breast cancer with palbociclib in combination with fulvestrant (estrogen receptor antagonist, treatment of HR^+^ metastatic breast cancer in postmenopausal women) and gorselin (agonist of luteinizing hormone-releasing hormone) (Table 2). The results revealed almost doubled median progression-free survival (9.5 vs. 5.6 months) and significantly increased objective response rate (25.0% vs. 11.1%) when compared to the endocrine monotherapy [43].

The primary objective of the PYTHIA clinical trial (Table 2) was to determine the association of progression-free survival with potential biomarkers for the selection of patients for the palbociclib–fulvestrant treatment. The results revealed a suppression of the serum thymidine kinase activity (83% of the patients exhibited a level below the detection limit on the 15th day of the therapy cycle). Higher serum thymidine kinase activity was associated with shorter progression-free survival [44]. Bagegni et al. [48] previously reported the reduction of the serum thymidine kinase activity after palbociclib treatment as a result of the NeoPalAna clinical trial (Table 3).

The efficacy and safety of the therapy as well as the patients’ quality of life depended on the palbociclib dosage method (standard: 3 weeks on/1 week off vs. continuous daily dose), and the accompanying endocrine therapy of choice was evaluated in the clinical trial NCT02630693 (Table 2). Dosage schedule had an influence on the toxicity profile, with higher rates of neutropenia at a continuous daily dosage.

The impact of palbociclib addition to the fulvestrant or fulvestrant and avelumab on patients whose disease progressed after previous CDK4/6 inhibitor plus endocrine therapy treatment was evaluated in the PACE clinical trial (Table 2). The results showed that the implementation of the palbociclib–fulvestrant combination therapy did not improve the progression-free survival (4.8 months for fulvestrant alone vs. 4.6 months for palbociclib–fulvestrant); however, additional immunotherapy with avelumab improved the progression-free survival up to 8.1 months [46].

Endocrine therapy may be ineffective in metastatic ER^+^ breast cancer due to the resistance arising from alterations in the fibroblast growth factor receptor pathway (FGFR, concerns about 15% of ER^+^ breast cancers). The main purpose of the NCT03238196 clinical trial (Table 2) was to determine the safety and tolerability of the three-component therapy: palbociclib–fulvestrant–erdafitinib. Median progression-free survival was 3 months, whereas for patients with high levels of FGFR amplification, it was higher (6 months). Adverse events included mucositis (67%), hyperphosphatemia (61%), neutropenia (47%), and anemia (29%) [47].

The efficiency of bazedoxifene, another endocrine therapy agent (estrogen-receptor agonist/antagonist), was evaluated in endocrine-resistant HR^+^ breast cancer patients during clinical trial NCT02448771 (Table 3). The promising results (median progression-free survival was 3.6 months) and the study’s limitations (small number of patients) highlighted the need for further evaluation of the palbociclib–bazedoxifene combination therapy [49].

The influence of a combination of hormonal (exemestane—aromatase inhibitor) and targeted therapy (everolimus—mTOR kinase inhibitor, palbociclib) was an investigational intervention evaluated in phase I/II NCT02871791 (Table 3) of the clinical trial for metastatic breast cancer resistant to CDK4/6 inhibitors. Although the clinical benefit rate did not meet the expectations (18.8%, prespecified threshold  ≥ 65%), the genomic and transcriptomic analysis of the biopsies enabled the identification of potential elements associated with acquiring resistance to the therapy [50].

CheckMate 7A8 study (Table 3) evaluated neoadjuvant treatment based on palbociclib with anastrozole (a non-steroidal aromatase inhibitor) and nivolumab in patients with ER^+^ breast cancer. This trial was terminated early due to grade 3/4 liver adverse events, apart from the ones mentioned previously. This trial was an important indication that such a combination of therapeutics should not be further evaluated [51].

The influence of the combination of palbociclib–anastrozole and palbociclib–giredestrant (nonsteroidal estrogen receptor agonist) was the main objective of the coopERA clinical trial (Table 3). The complete pathological response was similar between the two groups—4.6% for palbociclib–anastrozole and 4.5% for palbociclib–giredestrant—which is similar to the occurrence of grade 3–4 adverse events, such as neutropenia (27% and 26%, respectively) and decreased neutrophil count (15% for both) [52].

In the latest reviews concerning the clinical outcomes in HR^+^/HER^-^ breast cancer patients, Samjoo et al. [53] and Brain et al. [54] reported that patients’ life quality did not worsen after palbociclib treatment. Whereas Samjoo et al. [53] analyzed a broad population, including randomized control trials, single-arm clinical trials, and real-world evidence, Brain et al. [54] focused on the treatment of elderly people.

Although palbociclib is used primarily in the ER^+^/HER2^−^ breast cancer treatment, senescence and arrest of cell proliferation have also been explored for metastatic, triple-negative (TNBC, ER^−^/PR^−^/HER2^−^, PR—progesterone receptor) breast cancer treatment. In the Clinicaltrials.gov database, there are six investigations concerning the use of palbociclib in TNBC (Table 4). TNBC constitutes 12–17% of all breast cancers. They have a more aggressive phenotype, a higher proliferative index, and a worse prognosis than HR^+^ or HER2^+^ [55]. Because in TNBC there are no expressed HR or HER2 proteins, hormone therapy and HER2 targeted drugs are not efficient; therefore, the main therapeutic option is chemotherapy or chemotherapy combined with platinum-based compounds, such as carboplatin. Novel FDA-approved therapeutics for TNBC include poly adenosine diphosphate-(ADP)-ribose polymerase inhibitors (PARPi) and immunotherapy [56,57,58,59]. Because 51% of TNBCs express Rb, the application of CDK4/6 inhibitors as a therapy has a rational basis [60].

In preclinical research, palbociclib in combination with cisplatin exhibited a synergistic effect in MDA-MB-231 cells in prolonged sequential strategy in which the CDK4/6 inhibitor sensitized the cells to cisplatin. Importantly, the treatment of MDA-MB-231 cells with such a combination shows Rb dependence [61]. In another research, palbociclib, as a senescence inducer combined with navitoclax responsible for the removal of senescent cells, delayed the growth of tumor and reduced metastases in a mouse xenograft model of human TNBC [62]. There are ongoing clinical trials for the evaluation of the efficacy of palbociclib in combination with active compounds in TNBC with hyperactivated extracellular signal-regulated kinase and/or CDK4/6 (combined drug: binimetinib, NCT04494958) and androgen receptor positive TNBC (combined drug: avelumab, NCT04360941). The potential combination therapy strategies based on palbociclib for TNBC have been reviewed by Hu et al. [63].

In 2011, the clinical trial for advanced metastatic breast cancer (NCT01320592) started, where an admission criterion was retinoblastoma expression (Rb^+^) rather than the HR/HER2 status because of the capability of palbociclib to inhibit cell cycle through Rb-E2F blockade. In the said study, patients with advanced ER^+^/HER2^−^ overexpressing HER2 and TNBC were enrolled. The combination of paclitaxel (a cytostatic agent that targets microtubules) with palbociclib was evaluated for safety, tolerability, feasibility, and initial activity (33.3–60% response) and met initial expectations [64].

Patients with TNBC that expresses androgen receptor (AR^+^) participated in a clinical trial with a palbociclib–bicalutamide (androgen receptor inhibitor) combination as interventional therapy (NCT02605486, Table 4). The studies did not reveal unexpected toxicity and exhibited progression-free survival at 6 months in 11 participants.

TNBCs, which had not yet been treated, were the purpose of the clinical research conducted by Zhejiang Cancer Hospital (NCT03756090, Table 4). In this trial, dose-dense chemotherapy (epirubicin and cyclofosfamid, followed by paclitaxel) in combination with palbociclib was evaluated. The overall response rate, antitumor effect dependent on kinase profile, and safety were monitored in the clinical trial started in 2020 (PALBOBIN, Table 4). The dose-escalation study was carried out as part of the clinical trial PAveMenT (Table 4). Patients with TNBC were mainly treated with palbociclib in combination with immunotherapy, avelumab [65]. In 2022, a clinical trial of CAREGIVER (Table 4) began. In this investigation, the influence of carboplatin as a chemotherapeutic agent in combination with palbociclib will be evaluated.

**Table 4:** Selected clinical trials with therapy for TNBC based on palbociclib with other therapies according to the Clinicaltrials.gov database.

NCT Number	Phase	Status	Conditions	Sponsor/Study Start	Intervention	Literature
NCT01320592	I	completed	advanced ER^+^/HER2^−^ HER2 overexpressing and TNBC	Abramson Cancer Center at Penn Medicine/2011	paclitaxel, palbociclib	[64]
NCT02605486	I/II	active, not recruiting	postmenopausal AR^+^ TN MBC	Memorial Sloan Kettering Cancer Center/2015	bicalutamide, palbociclib	[66]
NCT03756090	not applicable	unknown	neoadjuvant therapy in TNBC	Zhejiang Cancer Hospital/2018	epirubicin, cyclofosfamid, paclitaxel, palbociclib, placebo	
NCT04360941 (PAveMenT)	I	recruiting	AR^+^ TNBC	Royal Marsden NHS Foundation Trust/2020	avelumab, palbociclib	[65]
NCT04494958 (PALBOBIN)	I/II	completed	TN ABC with ERK and CDK4/6 activation	Foundacion Oncosur/2020	binimetinib, palbociclib	
NCT05067530 (CAREGIVER)	II	not yet recruiting	untreated TNBC	Medical University of Gdansk/2022	paclitaxel, carboplatin, palbociclib	

TNBC, triple-negative breast cancer; Rb^+^, Retinoblastoma-expressing; AR, androgen receptor; TN MBC, triple-negative metastatic breast cancer; TN ABC, triple-negative advanced breast cancer.

#### 2.2.2. Solid Tumors

Patients with non-small-cell lung carcinoma (NSCLC) were subjected to palbociclib monotherapy (Table 5) that was well tolerated, and progression-free survival was comparable to other chemotherapeutic agents [67]. Additionally, preclinical studies conducted by Gopalan et al. [68] using palbociclib with different combinations revealed significant inhibition of cell growth in tumors with RAS mutations when palbociclib was applied with mTOR kinase (mammalian target of rapamycin) inhibitors, setting a new direction of research.

Evaluation of the influence of the combination mirdametinib (selective and non-ATP-competitive mitogen-activated protein kinase (MEK) inhibitor)–palbocilib for treatment of cancers with *KRAS* mutations, especially of lung origin, was the purpose of the NCT02022982 (Table 5) clinical trial. No results have been published for this trial so far.

Another trial (Table 5) evaluated the efficiency of mavelertinib (an epidermal growth factor receptor tyrosine kinase inhibitor) in NSCLC in combination with palbociclib. The study was terminated due to the decision to discontinue further development of mavelertinib [69].

Investigating the potency of targeted anticancer therapies for advanced cancers guided by the genomic alterations known as drug targets was the objective of a TAPUR clinical trial (Table 5). Among 17 different treatments, palbociclib was also tested. Among patients with advanced pancreatic or biliary cancer with *CDKN2A* (cyclin-dependent kinase inhibitor 2A) loss or mutation, palbociclib monotherapy did not demonstrate any clinical activity [70]. For patients with NSCLC (non-small-cell lung carcinoma) with *CDKN2A* alterations, palbociclib monotherapy demonstrated modest antitumor activity [71]. A TAPUR clinical trial among patients with soft tissue sarcoma with *CDK4* amplification met the expectations of the trial with a median progression-free survival of 16 weeks and a median overall survival of 69 weeks [72]. In Canada, a similar clinical trial (CAPTUR, Table 5) to monitor the changes in tumors after matching therapy, including palbociclib, based on genetic changes started in 2018.

The safety and recommended doses of palbociclib–cytotoxic agents were examined in a phase I dose-escalation study. The objective response rates were 12.5 and 25% for cisplatin and carboplatin, respectively. The results of the trial revealed an acceptable safety profile [73].

Two clinical trials (Table 5) evaluated the binimetinib (MEK inhibitor)–palbociclib combination. The first trial, started in 2017, examined dose escalation in patients with lung cancer (NSCLC with *KRAS* gene mutation). The second trial was conducted in conjunction with a coclinical trial with patient-derived xenografts. Although clinical evaluation of this therapeutic combination proved its safety and activity, treatment of patient-derived xenografts revealed a feedback activation of receptor tyrosine kinases and acquired resistance [74].

In combination with cetuximab, palbociclib is also being tested in a phase III clinical trial that will determine if the therapy improves the overall survival rate in patients with metastatic head and neck squamous cell carcinoma (ClinicalTrials.gov ID: NCT04966481). Promising results were also obtained in a phase II clinical trial of palbociclib in patients with metastatic colorectal cancer (ClinicalTrials.gov ID: NCT01037790) [75]. These studies were the starting point to test the CDK4/6 inhibitor in combination with immunotherapy (ClinicalTrials.gov ID: NCT03446157). Inhibition of CDK4/6 with palbociclib demonstrated single agent activity in patients with recurrent ovarian cancer in a phase II study of efficacy and safety (ClinicalTrials.gov ID: NCT01536743) [76]. Promising results, as manifested by an increased progression-free survival, were also obtained in a phase II clinical trial to evaluate the efficacy of the letrozole–palbociclib combination against metastatic ER^+^ endometrial cancer (ClinicalTrials.gov ID: NCT02730429) [77].

#### 2.2.3. Hematopoietic Cancers

Multiple myeloma, the second most common hematopoietic cancer, exhibits deregulation of CDK4/6 that appears as the loss of cell cycle control [78]. Preclinical data obtained with human multiple myeloma cell xenografts revealed that palbociclib induces prolonged early arrest of the G1 cell cycle phase. Furthermore, cells were sensitized to cytotoxic killing by bortezomib (a proteasome inhibitor) and dexamethasone (an anti-inflammatory glucocorticosteroid) [79,80,81]. In 2008, Pfizer decided to start a clinical trial (Table 6) with palbociclib in combination with bortezomib plus dexamethasone. The toxicity profile was consistent with known therapeutics, and the most commonly reported adverse event was thrombocytopenia. The results demonstrated an objective response in 20% of the patients and an additional stable disease in 44% [82].

In a terminated clinical trial performed by Pfizer in 2014 (Table 6), where dexamethasone and lenalidomide (an immunomodulatory agent) were planned as therapeutic agents together with palbociclib as an intervention, neither the efficiency nor the safety were insufficient; rather, low enrollment was observed [83].

The very early stage of clinical trials for palbociclib efficacy in hematological cancers constitutes NCT00420056 (Table 6) for patients with mantle cell lymphoma. The results confirmed the CDK4/6 inhibition caused by palbociclib by the observation of Rb phosphorylation. The median time to progression and progression-free survival were 4 months [84].

Since preclinical data suggested that palbociclib is capable of sensitizing mantle cell lymphoma cells, a clinical study has been conducted in which further killing with bortezomib was facilitated (Table 6). The trial was terminated due to myelosuppression, which was a dose-limiting factor; however, the studies allowed for the determination of a recommended dose for future studies [85].

To evaluate the efficacy of ibrutinib (Burton tyrosine kinase (BTK) inhibitor) and palbociclib combination in patients with mantle cell lymphoma, a phase II clinical trial started in 2018, based on the previously performed phase I (Table 6). During phase I, maximum tolerated doses of both therapeutics were determined based on the results of dose-limiting toxicity. Phase I revealed the overall and complete response rates of 67% and 37%, respectively [86].

The expression of c-Myb (a DNA-binding transcription factor) leads to the growth of leukemia by cyclin D3, CDK6, and Bcl-2. The transcriptional regulation of CDK6 and Bcl-2 (regulator proteins; block programmed cell death) influences the c-Myb transcription factor expression; thus, the CDK6 inhibitor may have an important influence on leukemia cells. The single-arm phase I dose-escalation study performed with the palbociclib–dexamethasone combination (Table 6) revealed a lack of dose-limiting toxicity. The results suggested the need for future trials combining palbociclib with newer agents [87]. Palbociclib in combination with other chemotherapeutics in a pilot study (ClinicalTrials.gov ID: NCT03792256) exhibited safety and good tolerability of use in children and young adults with relapsed/refractory acute lymphoblastic leukemia and lymphoma. Expansion of this trial in currently undertaken studies (ClinicalTrials.gov ID: NCT04996160) will reveal the feasibility and activity of the CDK4/6 inhibitor [88].

**Table 6:** Selected clinical trials with therapy concerning hematopoietic cancers based on palbociclib with other therapeutics according to the Clinicaltrials.gov database.

NCT Number	Phase	Status	Conditions	Sponsor/Study Start	Intervention	Literature
NCT00420056	I	completed	previously treated MCL	Pfizer/2007	palbociclib,	[84]
NCT00555906	II	completed	patients with MM after prior treatment	Pfizer/2008	bortezomib dexamethasone, palbociclib	[82]
NCT01111188	I	terminated	relapsed MCL	Weill Medical College of Cornell University/2010	bortezomib, palbociclib	[85]
NCT02030483	I	terminated	relapsed or refractory MM	Weill Medical College of Cornell University/2014	dexamethasone, lenalidomide, palbociclib	
NCT02159755	I	active, not recruiting	recurrent MCL	National Cancer Institute/2014	ibrutinib, palbociclib	[86]
NCT03478514	II	active, not recruiting	previously treated MCL	Alliance Foundation Trials, LLC/2018	ibrutinib, palbociclib	
NCT03472573	I	completed	relapsed or refractory BCALL	Sidney Kimmel Cancer Center at Thomas Jefferson University/2018	dexamethasone, palbociclib	[87]

MM, multiple myeloma; MCL, mantle cell lymphoma; BCALL, B-cell acute lymphoblastic leukemia.

The studies presented above are only some examples of clinical studies that reveal the potency of palbociclib as an effective anticancer agent. It is important to emphasize that the CDK4/6 inhibitor is most effective when the specific aberration of the cell cycle occurs, particularly in neoplastic cells, which highlights the value of the detailed determination of the nature of neoplastic changes in particular patients in the early stages of the disease. An important limitation of palbociclib that hinders the treatment is the dose-limiting toxicity of palbociclib as a monotherapy or in combination. The most frequently reported adverse events in breast cancer patients are neutropenia and gastrointestinal side effects that cause interruptions in palbociclib dose administration [89]. An additional challenge in using CDK4/6 inhibitors is that tumors may develop resistance to these treatments over time, which creates the need to establish alternative therapeutic strategies. Moreover, due to the kinase-independent activity of CDK6, eliminating the protein may be more advantageous than merely inhibiting its activity [14,15,27,28].

### 2.3. Resistance to Palbociclib

The main clinical problem in endocrine breast cancer therapy is an intrinsic or acquired (secondary) clinical resistance to CDK4/6 inhibitors, including palbociclib. Many resistance mechanisms have been proposed, including changes in cell metabolism, stromal function, cell cycle machinery, and increased activity through oncogenic growth factor signaling [90]. It is important to identify and predict the likelihood of resistance development or determine the presence of inherent resistance mechanisms. Possible pathways through which resistance can occur are abnormal activation of cyclin D–CDK4/6 or cyclin E–CDK2, loss of pRb, activation of the PI3K/AKT/mTOR pathway, activation of RAS, upregulation of c-Myc, or TK1 activity [91,92,93]. The crucial step before the initiation of CDK4/6 inhibitor therapy is to identify patients who exhibit primary resistance to inhibitors (approximately 10% of the patients) through an increase in TK1 activity or cyclin E1 expression. Currently, there is an ongoing observational clinical trial to identify and monitor the resistance to first-line breast cancer treatment with CDK4/6 inhibitors in combination with aromatase inhibitors (NCT04660435, TRIESIAS, Fondazione Sandro Pitigliani).

In the secondary resistance to CDK4/6 inhibitors, there is a significant importance of retinoblastoma expression that affects E2F activation and the cyclin E–CDK2 axis; therefore, there arises the independence of the cell cycle progression from the CDK4/6 pathway. According to the results presented by Condorelli et al. [94], malignant cells from breast cancer patients are able to avoid the effect of CDK4/6 inhibition through the retinoblastoma protein mutations after exposure to palbociclib and ribociclib. Therefore, it is crucial to choose alternative therapies in such cases. New targeted drugs as an addition to the therapeutic approach in order to overcome the resistance to CDK4/6 inhibitors are currently being explored. In clinical practice, therapeutic strategies are also employed after the emergence of resistance to CDK4/6 inhibitors, including switching to other CDK4/6 inhibitor–endocrine therapy combinations (palbociclib-resistant cells are also resistant to ribociclib but sensitive to abemaciclib [95]), combining targeted therapy, and switching to chemotherapy [92].

### 2.4. Palbociclib—The Molecule

**Figure 4:**
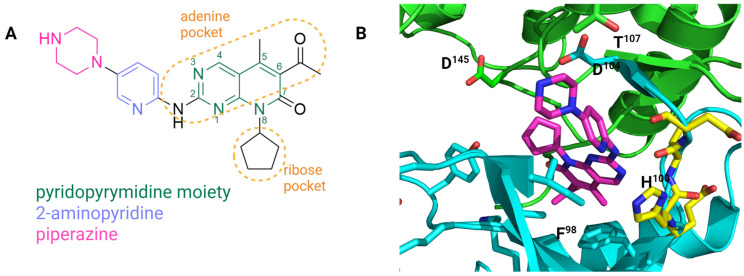
Structure of palbociclib (**A**) and its binding mode (**B**) to CDK6 (PDB ID: 5L2I). Magenta—palbociclib; cyan part of the CDK6 structure—N-lobe; cyan sticks—ATP binding site; green part of the CDK6 structure—C-lobe; hinge region—yellow sticks. Palbociclib regions responsible for interactions with the ribose pocket and the adenine pocket, based on Shan et al. [99]. Created in BioRender. Sienczyk, M. (2024); https://BioRender.com/b49h651 (accessed on 6 November 2024).

Palbociclib is a member of the pyridopyrimidines 2-{[5-(piperazin-1-yl)pyridin-2-yl]amino}pyrido[2,3-d]pyrimidin-7-one with methyl, acetyl, and cyclopentyl substituents at the C^5^, C^6^, and C^8^ positions, respectively (Figure 4A). The crystallographic data obtained by Chen et al. [24] revealed an inactive conformation of bilobal CDK6 in a complex with palbociclib (Figure 4B) localized in the ATP binding region between two lobes. Palbociclib interacts with the CDK6 hinge region Val^101^ by its 2-aminopyrimidine group that, together with N^3^, plays a fundamental role in binding (Figure 4B). Alkylation of the amino group of the 2-aminopyridine moiety results in loss of activity. The cycloalkyl substituent at position C^8^ of the pyridopyrymidine moiety interacts with the lipophilic subpocket similar to the acetyl at C^6^ position (hydrophobic back pocket) [96]. The piperazine ring of palbociclib is stabilized by Asp^104^ and Thr^107^ [24,97]. The gatekeeper Phe^98^ residue and the back pocket behind are close to the acetyl group at C^6^ position. As the molecular docking results obtained by Maganhi et al. [98] show, the interaction of palbociclib with CDK6 is based on a combination of hydrogen bonds and hydrophobic interactions that allow for conformational freedom at the piperazine ring. Since the piperazine moiety of palbociclib is exposed to the solvent, it is possible to introduce modifications in this region of the molecule.

As reported by Chen et al. [24], Thr^107^ is one of the selectivity determinants in CDK4/6. CDK1/2/3/5 have Lys at this position, which causes electrostatic repulsion of the palbociclib piperazine moiety. Chen et al. [24] also postulated, based on the sequence alignment between CDK4 and 6 and the crystallographic data obtained for CDK6–abemaciclib, that His^100^ located in the hinge region (which is present only in CDK4 and 6) is responsible for the palbociclib selectivity between CDKs. The specificity of palbociclib through interacting with CDK4/6’s conserved His^100^ was also described by Ris et al. [97].

**Figure 5:**
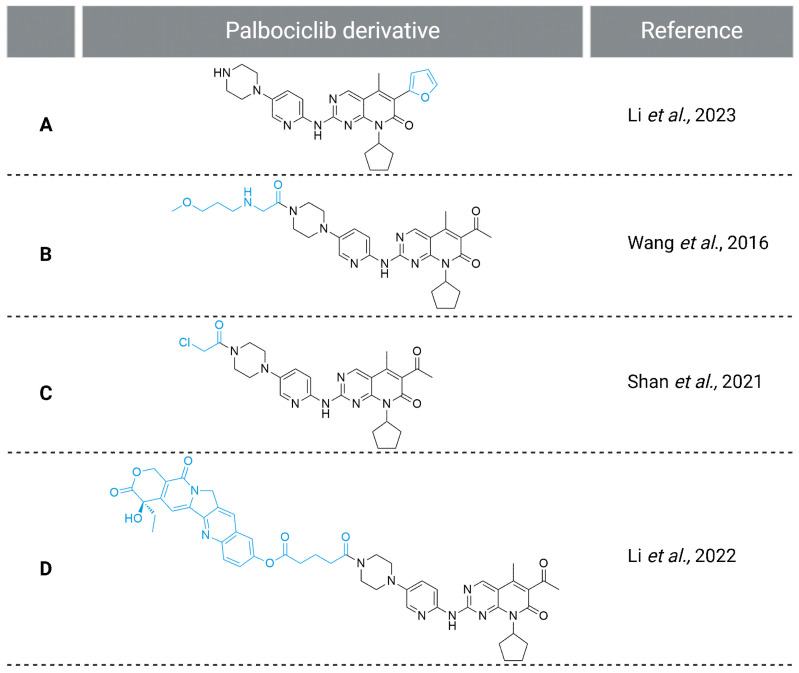
Palbociclib derivatives with antiproliferative potential [99,100,101,102]. Created in BioRender. Sienczyk, M. (2024); https://BioRender.com/y81q468 (accessed on 6 November 2024).

Based on the crystal structure of palbociclib and CDK6 (PDB ID: 5L2I, Figure 4), Li et al. [100] designed palbociclib analogs with aromatic groups introduced at the C^6^ position of the pyridopyrimidine moiety. They deduced that the substituent might occupy the back pocket and interact with Phe^98^, leading to increased activity against CDK6. A furyl derivative of palbociclib (Figure 5A) exhibited the highest potency against the MDA-MB-453 cell line (triple-negative breast cancer) cell line, preserved selectivity to CDK4/6, and exhibited better stability in the mouse liver microsome assay than palbociclib [100].

The functionalization of piperazine, the solvent-exposed moiety of palbociclib, by Wang et al. [101] resulted in the generation of a 3-methoxypropylamine derivative of palbociclib (Figure 5B) with significant cytotoxic activity in the MCF-7 breast cancer cell line and in vivo tumor growth inhibition in a breast cancer rat model. Based on the crystal structure of CDK6 with palbociclib (PDB ID: 2EUF), Shan et al. [99] observed the Thr^107^’s potential to be used as a nucleophile for the formation of a covalent bond with the palbociclib derivative. They designed and synthesized a series of palbociclib derivatives with electrophilic warheads and linkers suitable for piperazine nitrogen modification. In vitro studies revealed an intensified cytotoxic effect of the chloroacetylamide derivative of palbociclib (Figure 5C) in human breast cancer cell lines (MDA-MB-231 and MDA-MB-453) and human NSCLC cell line (H1299) when compared to palbociclib [99]. Li et al. [102] designed and synthesized double-target inhibitors, the structure of which includes palbociclib and 10-HCPT, which is an inhibitor of topoisomerase I. One of the compounds obtained with the succinic acid linker between the two inhibitors (Figure 5D) demonstrated a high potential for future development due to the improved antiproliferation effect and cell cycle arrest rate when compared to individual inhibitors in lung cancer cells. It is interesting that the cytotoxicity of the double-target inhibitor was lower than the topoisomerase inhibitor alone [102].

### 2.5. Palbociclib as a Building Part of CDK4/6 Degraders

**Figure 6:**
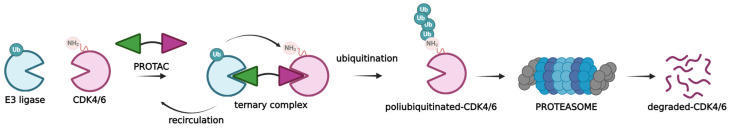
The principle of targeted protein degradation using CDK4/6-directed PROTACs [106]. Created in BioRender. Sienczyk, M. (2024); https://BioRender.com/o44i201 (accessed on 6 November 2024).

One of the new approaches designed to precisely degrade the protein of interest (POI) is proteolysis targeting chimeras (PROTACs). Described by Crews and Deshaies in 2001, the concept of PROTACs is based on the induction of ubiquitination followed by proteasomal degradation of POIs [103]. PROTACs are heterobifunctional molecules that contain a POI-specific ligand that is connected by a linker with a moiety characterized by the potency of recruiting E3 ligases (Figure 6) [104]. Compared to small-molecule drugs, the targeted protein degradation by PROTACs has some advantages, such as improved specificity and selectivity as well as the elimination of nonenzymatic proteins. Because binding events occur transiently, once the target POI undergoes ubiquitination and subsequent proteasomal degradation, PROTACs are then recycled. (Figure 6). Such recirculation could be beneficial for the dosage of the therapeutic, thus allowing for the limitation of dose-limiting toxicity [105].

The development of a novel strategy against CDK4/6-centered malignancies is of great interest and importance, and one of those strategies may be the application of PROTACs [13]. New opportunities provided by PROTACs enable E3 ligase to ubiquitinate the CDK4/6 (POI) targeted by a specific binder—palbociclib—followed by proteasomal degradation. This strategy may help overcome drug resistance through complete elimination of the CDK4/6. The palbociclib structure, especially the solvent-exposed piperazine moiety, is a good starting point for using this molecule as a structural element of chimeras.

**Figure 7:**
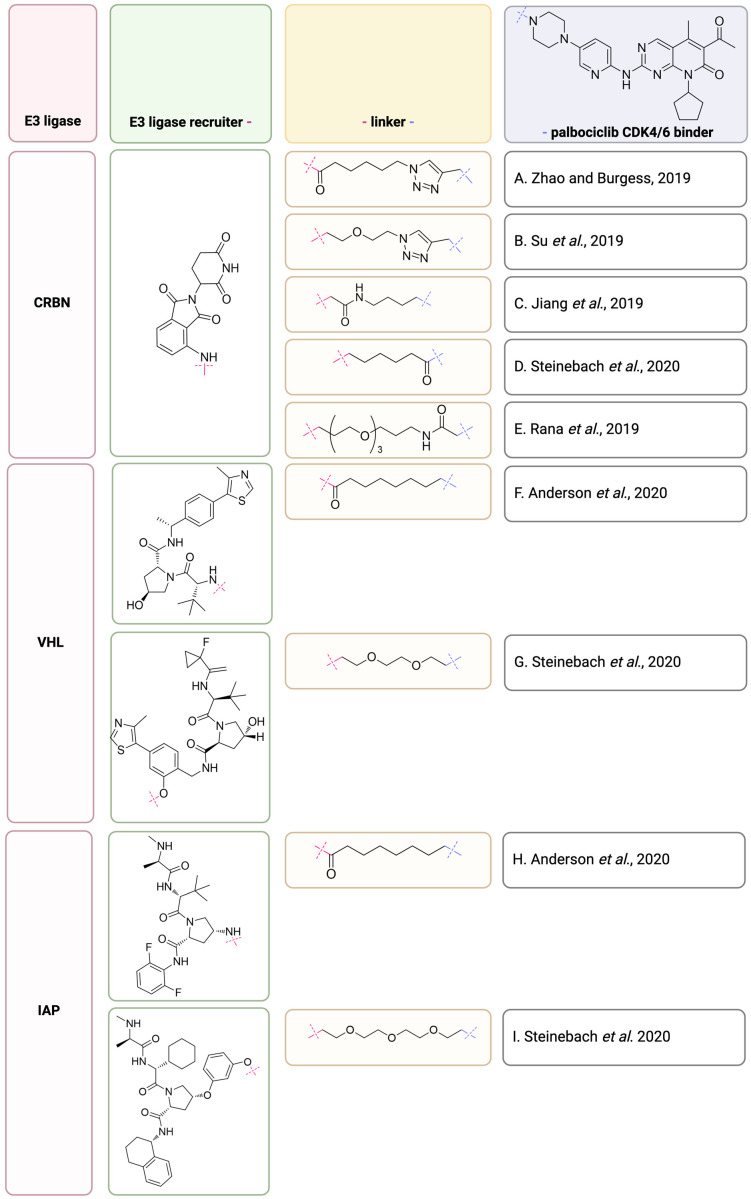
Examples of PROTAC compounds directed against CDK4/6 with various recruiters of E3 ligases [28,107,108,109,110,111]. Created in BioRender. Sienczyk, M. (2024); https://BioRender.com/q78b112 (accessed on 6 November 2024).

Several novel palbociclib-based CDK4/6 PROTACs have been developed in recent years. Zhao and Burgess [107] reported the first CDK4/6 PROTAC compound (Figure 7A) based on cereblon recruiter (pomalidomide) that was active against the MDA-MB-231 breast cancer cell line in the nanomolar range and reduced the pRB level. In their experiment with a triple-negative breast cancer cell line, they obtained an optimal CDK4 degradation in 4 h and a maximal decomposition of CDK6 in 6 h after treating the cells with 100 nM degrader concentration (Figure 7A) [107].

Su et al. [108] have reported a palbociclib-based PROTACs library based on pomalidomide as a ligase recruiter that contained structurally diverse linkers. They revealed that the obtained compounds selectively targeted CDK6. The most potent chimera (Figure 7B) inhibited the proliferation of several hematopoietic cancer cells (DC_50_ values for CDK6 and CDK4 were 2.1 nM and more than 150–180 nM, respectively). They also observed different responses to the selected PROTAC in various cell lines [108]. Jiang et al. [109] generated cereblon-recruiting degraders based on palbociclib, ribociclib, and abemaciclib and thus revealed that the specificity of the chimera against kinase depends on the kinase recruiter part of PROTAC and the character of the linker (length, composition). The obtained chimeras revealed important cellular effects, such as reduction in pRB and G1 arrest (treatment of Jurkat cells with 100 nM PROTAC shown in Figure 7C for 24 h induces G1 arrest). Furthermore, the selected compound (Figure 7C) exhibits an antiproliferative effect in mantle cell lymphoma cell lines (Granta-519 cells treated with 1 μM PROTAC from Figure 7C for 1 d resulted in the loss of CDK4/6) [109].

CDK6-selective degrader with cereblon as an E3-ligase (pomalidomide recruiter) was obtained by Rana et al. [110]. The designed chimera is characterized by a long linker moiety (Figure 7E). Selectivity is explained by the formation of a ternary complex. The inhibition assay performed in pancreatic MiaPaCa2 cells proved CDK6 reduction with the use of chimera (1 μM PROTAC presented in Figure 7E, 24 h), but no such effect was observed in cells treated with palbociclib (10 μM) or palbociclib with the PROTAC compound. The simultaneous involvement of CDK6 and cereblon was revealed when cereblon competition studies were performed [110].

Anderson et al. [111] obtained a series of PROTAC compounds based on palbociclib and the binders of three different E3-ligases: VHL (von Hippel-Lindau, Figure 7F), IAP (Inhibitor of Apoptosis Protein, Figure 7H), and cereblon. The preference for CDK6 degradation over CDK4 was confirmed not only for cereblon-directed PROTAC but also for VHL and IAP ligases in a dose–response degradation assay with Jurkat cell lines. After 24 h of treatment of Jurkat cells, they observed the degradation of CDK4 and CDK6 in a dose-dependent manner with pDC_50_ 7.6 and 8.4 for PROTAC from Figure 7F and 7.7 and 8.3 for PROTAC presented in Figure 7H [111].

In parallel, Steinebach et al. [28] developed PROTACs targeting four E3 ligases: cereblon, VHL, IAP, and MDM2 (mouse double minute 2). They obtained highly active, CDK-selective, VHL-recruiting PROTACs (“phenoxy”, example Figure 7G) in various cell lines that were able to inhibit cell proliferation more effectively than (human multiple myeloma, acute lymphoblastic leukemia) or comparably to (TNBC cell line) palbociclib. CDK 4 and CDK6 degradation was observed as that the remaining protein levels (%) after 16 h treatment at 0.1 μM concentration of the PROTAC presented in Figure 7G were 44 and 1.4, respectively. Cereblon-directed PROTAC (Figure 7D) developed by Steinebach et al. [28] could induce CDK6 degradation in a dose-dependent manner in murine cell lines. Similarly, CDK 4 and CDK6 degradation was observed as the remaining protein levels (%) after 16 h treatment at 0.1 μM concentration of the PROTAC presented in Figure 7D were 25 and 11, respectively. The MDM-directed PROTAC exhibits high lipophilicity, which is a cause for the low cell permeability of this compound. This is an important prerequisite for further studies of PROTAC structures to obtain beneficial physicochemical properties. They also designed and synthesized an IAP-directed chimera (Figure 7I) that induced CDK4/6 degradation in the multiple myeloma (MM.1S) cell line. CDK4 and CDK6 degradation was observed as the remaining protein levels (%) after 16 h treatment at 0.1 μM concentration of the PROTAC presented in Figure 7I were 77 and 75, respectively. Steinebach et al. [28] emphasized the importance of IAP-directed PROTAC compounds (SNIPERS, specific and nongenetic IAP-dependent protein erasers) that are capable of degrading not only the protein of interest (in this case CDK4/6) but also the IAP proteins themselves, which could facilitate the removal of malignant cells [28]. IAPs are known to be the key targets for cancer therapy. An increased expression of IAPs is observed in various malignancies, such as acute myeloid leukemia [112] and breast cancer [113], and can be poorly prognosed. Cellular degradation of IAPs may facilitate cell entry into apoptosis [106].

Recent advances in triple-negative breast cancer research revealed that inhibition of the CDK4/6 pathway with palbociclib in the triple-negative breast cancer cell line enhances the antiproliferative effect of cisplatin [61]. There are also attempts to apply PROTAC compounds as antiproliferative agents for triple-negative breast cancer. In 2023, Pu et al. [114] reported the chimera of palbociclib recruiter specific to CDK4/6 dependent on the DCAF16 (DDB1-and CUL4-associated factor 16) E3 ligase. The presented PROTAC that is based on KB02 ligand degrades CDK4/6, inhibits the proliferation of triple-negative breast cancer cells (MDA-MB-231), and exhibits therapeutic potential in the xenograft model in vivo. This research indicates a new field for PROTAC development with DCAF16 as an E3 ligase [114].

## 3. Discussion

Cyclin D–CDK4 and 6 are major actors in cell cycle regulation; therefore, targeting these proteins brings therapeutic benefits in neoplastic malignancies. One of the strategies is CDK4/6 inhibition through compounds that interact with the ATP binding pocket of the kinases mentioned above (Figure 4). Low molecular weight inhibitors of CDK4/6, such as palbociclib, ribociclib, and abemaciclib, are currently used in selected cancer diseases (e.g., breast cancer). Incorporation of CDK4/6 inhibitors in endocrine therapy as a treatment of metastatic breast cancer became an FDA-approved gold standard and is the most well-known use of these inhibitors. The most recent studies include a comparative evaluation of progression-free survival, overall survival, safety, and tolerability in combination with endocrine therapy (ER+/HER2- breast cancer treatment), palbociclib, ribociclib, and abemaciclib [115,116,117,118]. CDK4/6 inhibitors in combination with endocrine therapy elongate progression-free survival by two-fold. Cejuela et al. [117] indicate that abemaciclib reduces the risk of disease progression more effectively than others in refractory endocrine disease; the authors of the study also note that the results used to obtain the conclusions come from one center, and further evaluation is necessary (MONARCH 2 clinical trial, ClinicalTrials.gov ID: NCT02107703). They also note that palbociclib could be more beneficious for patients with drug–drug interactions, where an important issue is to avoid diarrhea or where poor tolerability is likely to occur [117]. Desnoyers et al. [116] reported that the most noticeable differences as far as drug therapy was concerned were observed not so much in efficacy but rather in safety and tolerability [116]. Palbociclib revealed a more frequent hematological toxicity and less gastrointestinal toxicity when compared to ribociclib and abemaciclib [115]. Danish studies performed on retrospective data did not indicate significant differences between the inhibitors in terms of overall survival, attesting ribociclib as the inhibitor with the longest median overall survival in first- and second-line patients. Furthermore, they described ribociclib and abemaciclib as inhibitors with significantly prolonged progression-free survival [118].

Preclinical and clinical approaches that utilized palbociclib as a therapeutic agent (alone or in combination) showed that this molecule could be employed in cancer treatment. In parallel, results showed that palbociclib has its limitations and is difficult in application. Improvements in CDK4/6 inhibitors’ selectivity, activity, and toxicity could boost their therapeutic potential. Overcoming drug resistance and the activity of non-kinase functions of CDK4/6 is essential in the clinic.

The first PROTAC molecules that entered the clinical trial testing were bavdegalutamide (ARV-110), PROTAC AR (androgen receptor) degrader, and vepdegestrant (ARV-471), PROTAC ER degrader, in ER+/HER− advanced/metastatic breast cancer [119]. The first-in-human phase I/II study (ClinicalTrials.gov ID: NCT04072952, Arvinas Estrogen Receptor, Inc., Pfizer) revealed the clinical safety and activity of vepdegestrant monotherapy in breast cancer patients. Furthermore, the degrader activity was also evaluated in combination with palbociclib, which resulted in greater tumor growth inhibition in xenograft models than palbociclib–fulvestrant [120]. There is currently an ongoing VERITAC-2 phase III clinical trial (ClinicalTrials.gov ID: NCT05654623, Pfizer), which is intended to indicate the effectiveness of vepdegestrant versus fulvestrant in people with metastatic breast cancer who were previously treated with endocrine-based therapy [121]. Additionally, vepdegestrant receives FDA Fast Track Designation as a single agent for the treatment of patients with metastatic breast cancer in 2024. Although palbociclib is involved in the previously mentioned clinical trials as a combination with a degrader compound, to our knowledge there are no clinical trials involving PROTACs that utilize palbociclib as a CDK4/6 binder.

As mentioned in Section 2.5, palbociclib can be incorporated in the structure of PROTAC compounds via the piperazine moiety. This region of the molecule, according to the crystallographic data, is solvent exposed; thus, it is available for incorporation into the palbociclib structure linker followed by the E3 recruiter. Zhao and Burgess reported the first palbociclib-based PROTAC that was active against a triple-negative breast cancer cell line with more potency than ribociclib-based chimera [107]. The possibility of selective CDK6 degradation by palbociclib-based PROTAC with pomalidomide as an E3 ligase recruiter was shown in 2019 by Su et al. [108] in their in cellulo assays. Taking into consideration the possibilities of structure modification, many research fields are open. From the research performed with pomalidomide as an E3 ligase recruiter in palbociclib-based PROTACs, we can conclude a significant impact of linker moiety that could be incorporated into the chimera structure [28,107,108,109,110]. Another option for PROTAC development is to choose the efficient E3 ligase recruiter [111].

## 4. Future Directions

Classic small-molecule drugs interact with the active site pockets of the target proteins, exerting their effects. What limits their application are genetic mutations that lead to changes in protein conformation or compensatory overexpression of target proteins that increase the risk of acquired drug resistance [106,122]. Such mutations resulting in a loss of activity of small-molecule inhibitors or resistance to inhibitors for cyclin-dependent kinase 4/6 (CDK4/6) have been reported [90]. In hematopoietic malignancies, breast cancer, and melanoma, CDK4 and 6 are considered attractive therapeutic targets. Although small-molecule inhibitors targeting CDK4/6 revolutionized breast cancer therapy and improved hematological malignancy therapies, the drawbacks of their use, such as developing resistance, became apparent [6]. Another important characteristic of CDK6 is its non-kinase activity; hence, silencing the protein is more valuable than inhibition of its activity [27,28]. Thus, the development of a novel strategy against CDK6-centered malignancies is of great interest and importance. The nature of PROTACs makes these compounds large in size and places them beyond Lipinski’s rule of five. Three main barriers that degraders have to overcome are chemical stability, solubility, and cell membrane permeability [123]. However, the past few years of PROTAC studies (examples in Figure 7) have revealed their selectivity and surprising pharmaceutical properties [124]. It could be advantageous to use a double knockdown process, which may be possible with the use of an E3 ligase-recruiting IAP-binding molecule, such as Smac/DIABLO mimetics that effectively interact with IAPs. As an example, the N-terminal Smac/DIABLO protein analogs, such as LCL161, MV1, or A410099, could be considered [106].

## Figures and Tables

**Table 1:** Selected clinical trials with therapy related to HR^+^/HER2^−^ breast cancer based on palbociclib and letrozole according to the Clinicaltrials.gov database.

NCT Number	Phase	Status	Conditions	Sponsor/Study Start	Intervention	Literature
NCT00721409 (PALOMA-1/TRIO-18)	I/II	completed	ER^+^/HER2^−^ ABC	Pfizer/2008	letrozole, palbociclib	[33]
NCT01684215	I/II	completed	Japanese postmenopausal women with ER^+^/HER2^−^ ABC	Pfizer/2012	letrozole, palbociclib	[36,37]
NCT01740427 (PALOMA-2)	III	completed	postmenopausal women with ER^+^/HER2^−^ ABC	Pfizer/2013	letrozole, palbociclib, placebo	[34]
NCT02297438 (PALOMA-4)	III	active, not recruiting	Asian postmenopausal women with ER^+^/HER2^−^ ABC	Pfizer/2015	letrozole, palbociclib, placebo	[35]
NCT02296801 (PALLET)	II	completed	ER^+^ EBC	NSABP Foundation Inc./2015	letrozole, palbociclib	[38]
NCT02600923	III	completed	postmenopausal women with HR^+^/HER2^−^ BC	Pfizer/2015	letrozole, palbociclib	[39]
NCT02679755	IV	completed	postmenopausal women with HR^+^/HER2^−^ BC	Pfizer/2016	letrozole, palbociclib	[40]
NCT02764541 (PELOPS)	II	active, not recruiting	HR^+^ early-stage BC	Dana-Farber Cancer Institute/2016	letrozole, tamoxifen, palbociclib	
NCT02907918 (PALTAN)	II	terminated	HR^+^/HER2^+^ early-stage BC	Washington University School of Medicine/2017	letrozole, trastuzumab, gorselin, palbociclib	[41]
NCT03633331	II	active, not recruiting	HR^+^/HER2^−^ MBC	Alliance for Clinical Trials in Oncology/2018	letrozole, fulvestrant, palbociclib	[42]

BC, breast cancer; ABC, advanced breast cancer; EBC, early breast cancer; MBC, metastatic breast cancer.

**Table 2:** Selected clinical trials with therapy related to HR^+^/HER2^−^ breast cancer based on palbociclib and fulvestrant according to the Clinicaltrials.gov database.

NCT Number	Phase	Status	Conditions	Sponsor/Study Start	Intervention	Literature
NCT01942135 (PALOMA-3)	III	completed	HR^+^/HER2^−^ ABC	Pfizer/2013	fulvestrant, gorselin, palbociclib placebo,	[43]
NCT02536742 (PYTHIA)	II	completed	postmenopausal women with HR^+^/HER2^−^ MBC	ETOP IBCSG Partners Foundation/2016	fulvestrant, palbociclib	[44]
NCT02630693	II	completed	ER^+^/HER2^−^ MBC	Canadian Cancer Trials Group/2016	fulvestrant, palbociclib, tamoxifen	[45]
NCT03147287 (PACE)	II	active, not recruiting	HR^+^/HER2^−^ MBC that stop responding for palbociclib and endocrine therapy	Dana-Farber Cancer Institute/2017	fulvestrant, avelumab, palbociclib	[46]
NCT03238196	I	active, not recruiting	ER^+^/HER2^−^/FGFR ^a^ MBC	Vanderbilt-Ingram Cancer Center/2017	fulvestrant, erdafitinib, palbociclib	[47]

BC, breast cancer; ABC, advanced breast cancer; EBC, early breast cancer; MBC, metastatic breast cancer; FGFR, fibroblast growth factor receptors; ^a^ amplified.

**Table 3:** Selected clinical trials with therapy concerning HR^+^/HER2^−^ breast cancer based on palbociclib with other therapeutics according to the Clinicaltrials.gov database.

NCT Number	Phase	Status	Conditions	Sponsor/Study Start	Intervention	Literature
NCT01723774 (NeoPalAna)	II	active, not recruiting	HR^+^/HER2^−^ BC	Washington University School of Medicine/2013	anastrozole, gorselin,	[48]
NCT02448771	I/II	completed	endocrine resistant HR^+^/HER2^−^ ABC	Dana-Farber Cancer Institute/2015	bazedoxifene, palbociclib	[49]
NCT02871791	I/II	completed	CDK4/6 inhibitor-resistant HR+/HER2^−^ MBC	Dana-Farber Cancer Institute/2016	everolimus, exemestane, palbociclib	[50]
NCT04075604 (CheckMate 7A8)	II	completed	postmenopausal women and men ER^+^/HER2^−^ PBC	Bristol-Myers Squibb/2019	nivolumab, anastrozole, palbociclib	[51]
NCT04436744 (coopERA)	II	completed	postmenopausal women HR^+^/HER2^−^ EBC, untreated	Hoffmann-La Roche/2020	giredestrant, anastrozole, palbociclib	[52]

BC, breast cancer; ABC, advanced breast cancer; EBC, early breast cancer; MBC, metastatic breast cancer; and PBS, primary breast cancer.

**Table 5:** Selected clinical trials with therapy concerning solid tumors based on palbociclib with other therapeutics according to the Clinicaltrials.gov database.

NCT Number	Phase	Status	Conditions	Sponsor/Study Start	Intervention	Literature
NCT01291017	II	completed	previously treated advanced stage IV NSCLC with Rb^wt^ and inactive (CDK)N2A	University of Florida/2011	palbociclib	[67,68]
NCT02022982	I	completed	NSCLC with *KRAS* mutation	Dana-Farber Cancer Institute/2014	mirdametinib, palbociclib	
NCT02349633	I/II	terminated	NSCLC	Pfizer/2015	mavelertinib, avelumab, palbociclib	[69]
NCT02693535 (TAPUR)	II	recruiting	patients with advanced tumors with gene alterations suitable for the mechanism of the therapy	American Society of Clinical Oncology/2016	in general: FDA-approved targeted anticancer drugs prescribed for treatment of patients with advanced cancer; palbociclib monotherapy	[70,71,72]
NCT02897375	I	completed	patients with advanced solid malignancies	Emory University/2016	carboplatin, cisplatin, palbociclib	[73]
NCT03170206	I	active, not recruiting	advanced NSCLC with *KRAS* gene alteration	Dana-Farber Cancer Institute/2017	binimetinib, palbociclib	
NCT03297606 (CAPTUR)	II	recruiting	patients with advanced tumors with gene alterations suitable for the mechanism of the therapy	Canadian Cancer Trials Group/2018	in general: commercially available targeted agents	
NCT03981614	II	active, not recruiting	colorectal cancer with *KRAS* and *NRAS* alteration	Academic and Community Cancer Research United/2019	binimetinib, palbociclib, trifluridine, and tipiracil hydrochloride	[74]

NSCLC, non-small-cell lung cancer; SCLC, small-cell lung cancer; Rb^wt^, wildtype retinoblastoma protein; (CDK)N2A, cyclin dependent kinase N2A; BC, breast cancer; OC, ovarian cancer; PC, prostate cancer.
